# Species-specific responses of invasive plants to parasitism are modified by climate warming

**DOI:** 10.1093/aobpla/plag015

**Published:** 2026-02-27

**Authors:** Chaonan Cai, Yongge Yuan, Dan Wu, Junmin Li

**Affiliations:** School of Advanced Study, Institute of Global Change and Evolutionary Ecology, Taizhou University, No. 1139 Shifu Road, Jiaojiang District, Taizhou, Zhejiang 318000, China; Zhejiang Key Laboratory for Restoration of Damaged Coastal Ecosystems, School of Life Sciences, Taizhou University, No. 1139 Shifu Road, Jiaojiang District, Taizhou, Zhejiang 318000, China; Taizhou Industrial Wastewater COD Deep Removal Key Laboratory, Taizhou Research Institute, Southern University of Science and Technology, No. 638 Donghuan Road, Jiaojiang District, Taizhou, Zhejiang 318000, China; College of Life and Environmental Sciences, Hangzhou Normal University, No. 2138 Yuhangtang Road, Hangzhou, Zhejiang 311121, China; Department of Science Education, Taizhou University, No. 1139 Shifu Road, Jiaojiang District, Taizhou, Zhejiang 318000, China; School of Advanced Study, Institute of Global Change and Evolutionary Ecology, Taizhou University, No. 1139 Shifu Road, Jiaojiang District, Taizhou, Zhejiang 318000, China; Zhejiang Key Laboratory for Restoration of Damaged Coastal Ecosystems, School of Life Sciences, Taizhou University, No. 1139 Shifu Road, Jiaojiang District, Taizhou, Zhejiang 318000, China

**Keywords:** biological invasion, *Cuscuta gronovii*, global warming, invasive species, parasitism, species-specific response

## Abstract

Parasitic plants can inflict significant damage to invasive plants and are considered biocontrol agents. Climate warming can affect the fitness of invasive plants and the efficacy of their biocontrol agents. However, the impact of climate warming on the responses of invasive plants to parasitism remains inadequately explored. To investigate this critical issue, we conducted a controlled warming experiment to assess the impact of a constant, continuous +2 °C temperature increase, consistent with projected global warming scenarios reported by the Intergovernmental Panel on Climate Change, on the responses of two common invasive plants in China, *Solidago canadensis* and *Bidens pilosa* to the parasitic plant *Cuscuta gronovii*. Our findings indicate that parasitism significantly reduces the growth of both invasive species irrespective of temperature increases. A significant interaction was observed between *Cuscuta* parasitism and the different host species, particularly affecting stem diameter, plant height, and root-to-shoot ratio of the host plants. Interestingly, while increased temperature independently did not significantly impact total biomass, aboveground biomass, or leaf number of the host plants, it exhibited marginal interactions with parasitism and the different hosts regarding belowground biomass of the hosts. Moreover, *C. gronovii* biomass was significantly influenced by host type; however, increased temperatures did not significantly affect the biomass of *C. gronovii* or its deleterious effects on host plants. Overall, these findings highlight the complex interplay among parasitism, host species, and environmental factors, which are crucial for comprehensively understanding invasive species dynamics and their ecological implications.

## Teaser

Parasitic plants are increasingly considered as biological control agents against invasive species, yet their effectiveness under climate warming remains uncertain. Using a controlled warming experiment, we show that a 2 °C temperature increase does not weaken the overall suppressive effects of parasitism but alters them in a species-specific manner. These findings highlight that climate warming can reshape host–parasite interactions, with important implications for predicting and managing biological invasions under future climate scenarios.

## Introduction

Climate change is a significant factor in global environmental changes. It impacts biodiversity, ecosystem functions, and human life quality, thereby often causing serious economic or ecological hazards (Dukes and Mooney 1999, Walther et al. 2009, Bradley et al. 2010, van Kleunen et al. 2018, Pyšek et al. 2020, Kumar Rai and Singh 2020, Bao et al. 2022). Scientific evidence indicates that global warming will increase for several decades (Meinshausen et al. 2009, 2022, IPCC 2021), with a projected 1.5–2°C increase by the mid-21st century under moderate emission scenarios. This temperature change will profoundly influence plant growth, including physiological, phenological and other traits, and plant interactions, especially between native and invasive species (Fang et al. 2021, Ren et al. 2021, 2022). Biocontrol has been proven to be an effective way of mitigating plant invasions (Sun et al. 2022). Biocontrol success can be influenced by multilayered interactions between invasive plants and the biotic and abiotic constituents of the invaded habitats in different climatic scenarios. Understanding the potential influence of a + 2°C temperature increase (a scenario consistent with IPCC 2021 projections and widely adopted in global change research) on the effectiveness of biocontrol efforts is essential for developing sustainable strategies for managing invasive species.

Previous studies have explored the potential effects of global warming on invasive plants, including altered distribution patterns, growth rates, competitive interactions, and augmented invasiveness (Dukes and Mooney 1999, Hellmann et al. 2008, Walther et al. 2009, Bradley et al. 2010). Recent studies indicate that increased temperatures due to climate change could hinder the spread of invasive plants (Jabran and Dogan 2020, Ren et al. 2021, 2022, Bao et al. 2022). On the contrary, global warming can enhance invasive plants’ growth and interspecific competitiveness, potentially due to increased soil organic matter mineralization and nutrient availability (Shaver et al. 2000, Rustad et al. 2001, Lu et al. 2013).

Invasive plants often interact with various organisms upon invading a new habitat, including parasitic plants, herbivores, and soil microbiota. The interactions between parasitic plants and their hosts often parallel those between herbivores and their hosts (Pennings and Callaway 2002). For example, they exhibit host preferences, decrease host biomass and change host allocation patterns, modify plant community structure and dynamics, and mediate interactions between host plants and other organisms (Pennings and Callaway 2002). Global warming may alter the biotic interactions within an ecosystem. For example, warming changes the beetle’s life history and metabolic rate of invasive *Ambrosia artemisiifolia*, reducing its biocontrol efficacy (Sun et al. 2022). Elevated temperatures affect the life history of herbivores and their metabolic rates (Bale et al. 2002, Hegland et al. 2009, Yang and Rudolf 2010, Hamann et al. 2021). It can also alter plants' physiological traits or chemical composition, potentially leading to changes in herbivore populations or shifts in the types of herbivores present, thereby altering the interactions between plants and herbivores (Rustad et al. 2001, Zvereva and Kozlov 2006, Hudson et al. 2011, DeLucia et al. 2012, Jamieson et al. 2017, Descombes et al. 2020, Maynard et al. 2022). Although the broader impacts of climate change on plant–herbivore interactions have been studied more frequently (Sun et al. 2020, 2022), the effects on host–biocontrol agent dynamics, particularly those involving parasitic plants such as *Cuscuta*, remain insufficiently explored. This knowledge gap highlights the need for further research into how global warming could affect the interactions between invasive plants and their biological control agents.

*Cuscuta* spp., as holoparasites, obtain essential resources, such as nutrients, water, and organic compounds, from their hosts through haustoria, consequently hampering the hosts’ growth (Pennings and Callaway 2002, Press and Phoenix 2005, Yang et al. 2019, Yuan and Li 2022). Previous studies have shown that parasitism adversely impacts the growth of invasive species, and parasitic plants could be exploited as prospective biocontrol agents (Pennings and Callaway 2002, Press and Phoenix 2005, Yu et al. 2009, Li et al. 2012, 2019, Těšitel et al. 2020; Gao et al. 2021, Cai et al. 2023). Furthermore, the impact of parasitism on invasive plants can be further influenced by other abiotic and biotic factors, such as soil microorganisms (Li et al. 2019) and nutrient availability (Gao et al. 2021). However, to date, no studies have examined whether elevated temperatures can influence the effectiveness and properties of parasitism on invasive plants.

In this study, we carried out a factorial experiment to investigate the effects of *Cuscuta* parasitism and elevated air temperatures on the growth of two invasive species. We aimed to address the following questions: (i) How do elevated temperatures influence the growth of parasitic plants and their host species? (ii) Will the detrimental effects (DEs) exerted by parasitic plants be exacerbated under elevated temperatures? (iii) Do the effects of warming and parasitism on host species vary with host species? The findings could provide significant theoretical insights and practical applications for the management of invasive plants and their associated parasitic weed *Cuscuta* spp. Understanding these dynamics is essential for developing effective strategies to mitigate the ecological impacts of invasive species in the context of a changing climate characterized by global warming.

## Material and methods

### Study species and soil substrate

We investigated two invasive plant species in China, *Solidago canadensis*, and *Bidens pilosa*, as host plants and the stem holoparasite *Cuscuta gronovii*, as the parasitic plant. *S. canadensis* is a perennial forb native to North America, widely naturalized in China since its introduction in the 19th century (Jiangsu Institute of Botany 1982, Zheng 1993). *B. pilosa* is an annual herb native to Central America, becoming invasive in eastern, southern, central, and southwestern China (Li et al. 2024). Based on our field investigation, *C. gronovii* can parasitize on both *S. canadensis* and *B. pilosa* and heavily suppress their growth (Yuan et al. 2023). Seeds of *S. canadensis* and *B. pilosa* were collected from a field near Taizhou University, Zhejiang Province, China (28°39′N, 121°23′E). Stems of *C. gronovii* were collected from a field near Taizhou University.

### Experimental set-up

The soil substrate was collected from land on a mountain devoid of invasive plants in the Jiaojiang District, Taizhou, China. The soil had a pH of 4.53, 11.71 g kg^−1^ organic matter, 0.280 g kg^−1^ total nitrogen, and 1.206 g kg^−1^ total phosphorus. The collected field soil was subjected to air drying, followed by thorough mixing and sieving through a metal grid with a mesh size of 1 cm before being placed into pots (diameter: 11 cm, height: 10 cm).

The experiment was conducted in the outdoor research garden on the Jiaojiang Campus, Taizhou University, Zhejiang Province, China (28°65′ N, 121°39′ E). The experiment was carried out in four replicated blocks (6.1 m × 2.1 m, length × width), and each block contained two triangular plots (sides of 2.5 m) spaced 1.1 m apart. One plot was artificially heated 24 h a day using three infrared heater lamps to simulate elevated temperature conditions (Model LPR2420/1500-L, Longpro Co., Ltd., Guangzhou, China), while the other plot was not heated. In each plot, 12 pots (height × diameter, 10 cm × 11 cm) filled with 1.5 kg of soil were randomly allocated. Six of the pots contained parasitized or non-parasitized *S. canadensis*, and the remaining six pots contained parasitized or non-parasitized *B. pilosa*, resulting in a total of 96 pots (4 replicated blocks × 2 plots × 2 species × 2 parasitism × 3 replicates).

On 8 May 2024, seeds of *S. canadensis* and *B. pilosa* were surface sterilized with 5% sodium hypochlorite for 15 minutes and then germinated in peat moss (QTS 0–6 + 10% Black Peat, NPK1, micro50, WA, pH5.5–6.5) in the greenhouse facilities of Taizhou University. After 25 days, two seedlings with comparable growth were selected and individually transplanted into separate pots. If any seedlings died within 1 week after the initial transplanting, they were replaced with similarly sized seedlings. Finally, the seedlings with the most similar size and growth status were retained for the subsequent treatments. On 4 July 2024, elevated temperature and parasitism treatments were initiated on the invasive plants. A 15-cm-long stem piece of *C. gronovii* was wound counterclockwise around the stems of host plants. Non-parasitized invasive plants were used as controls. In the elevated temperature treatments, continuous heating was applied with an LPR2420/1500-L infrared radiator at the Global Warming Experimental Park at Taizhou University, roughly increasing the air temperature by 2°C over natural ambient temperature variation. This warming treatment was chosen based on temperature change projections from previous studies (Meinshausen et al. 2009, IPCC 2021) and was consistently maintained until 9 August 2024. To ensure consistent soil moisture, water was sprayed daily onto the soil of each container. Additionally, 1 × Hoagland nutrient solution (100 ml) was applied weekly to each pot until the harvest of the materials to ensure proper plant growth and eliminate any impact of soil nutrient deficiencies.

### Harvest and measurements

Seventy days after transplanting, *C. gronovii* were harvested by carefully separating them from the host plant. Specifically, fine-tipped forceps were used to manually peel the stems of *C. gronovii* from the stems and leaves of host plants, with the separation process initiated at the attachment points of haustoria. The leaf number of the host plant was counted. The plant height and stem diameter were measured using a ruler and a vernier caliper, respectively. The host plants were subsequently divided into leaves, roots, and stolons, and each part was carefully placed inside separate paper bags. Following this, the different plant tissues were dried at 65°C in an oven until they reached a constant weight and then weighed to determine the dry biomass.

### Statistical analyses

To test the effects of elevated air temperature, parasitism, host plant species, and their interactions on the growth of host plants, a linear mixed model was implemented using the *lmer* function in the R package *lme4*, which utilizes maximum likelihood (ML) to estimate model parameters (Bates et al. 2014). The models were constructed using elevated temperature, host species, parasitism, and their interactions as fixed factors and block/plot as random factors. The growth variables included total biomass, aboveground biomass, belowground biomass, leaf number, stem diameter, and plant height. Additionally, a linear mixed model was constructed to evaluate the effects of elevated temperatures, plant species, and their interaction on *C. gronovii* biomass. The model included warming, host plant species, and their interaction as fixed factors, with block/plot serving as the random factor. The DEs on hosts were calculated as the difference in biomass between the parasitized plants and the mean biomass of the control plants, standardized to the mean biomass of the control plants (Li et al. 2012). Belowground biomass, aboveground biomass, and total biomass, along with plant height, leaf number, and stem diameter, were tested. The effect of elevated temperature on DE was assessed using a one-way ANOVA with significance at *P* < .05. According to the results of one-sample *t*-test, DE > 0 indicates that parasitism facilitated host growth, DE < 0 indicates that parasitism suppressed host growth, and DE = 0 indicates that parasitism did not affect host growth (Li et al. 2012). The normality of variance of the residuals was assessed prior to the analyses. To ensure normality and equal variance, the leaf number data were square-root transformed, and the root:shoot ratio data were log-transformed. A Fisher’s least significant difference (LSD) was used to evaluate differences among treatments at a 5% significance level. All the statistical analyses were performed using R software version 4.3.0 (R Core Team 2023).

## Results

### Effects of parasitism, host species, and elevated temperatures on the growth of invasive plants

**Figure 1:**
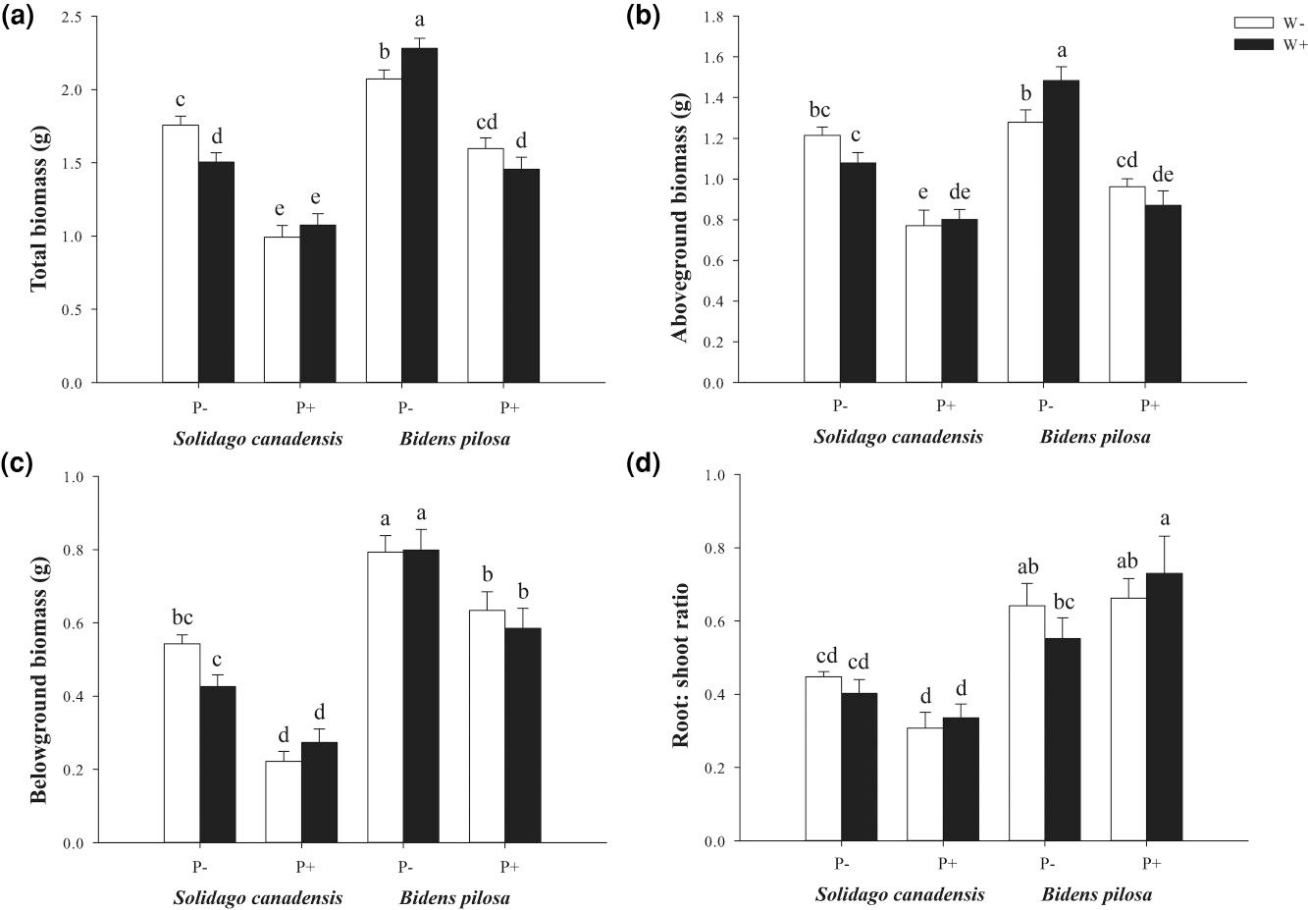
Effect of warming and *C. gronovii* parasitism on the (a) total biomass, (b) aboveground biomass, (c) belowground biomass, (d) root:shoot ratio of the invasive plant species *Solidago canadensis* and *Bidens pilosa*. Values are means ± SE. Different letters indicate significant differences among treatments based on LSD *post hoc* tests (*P* < .05). W+: warming present, W−: warming absent, P+: parasitized by *C. gronovii*, P−: non-parasitized.

**Figure 2:**
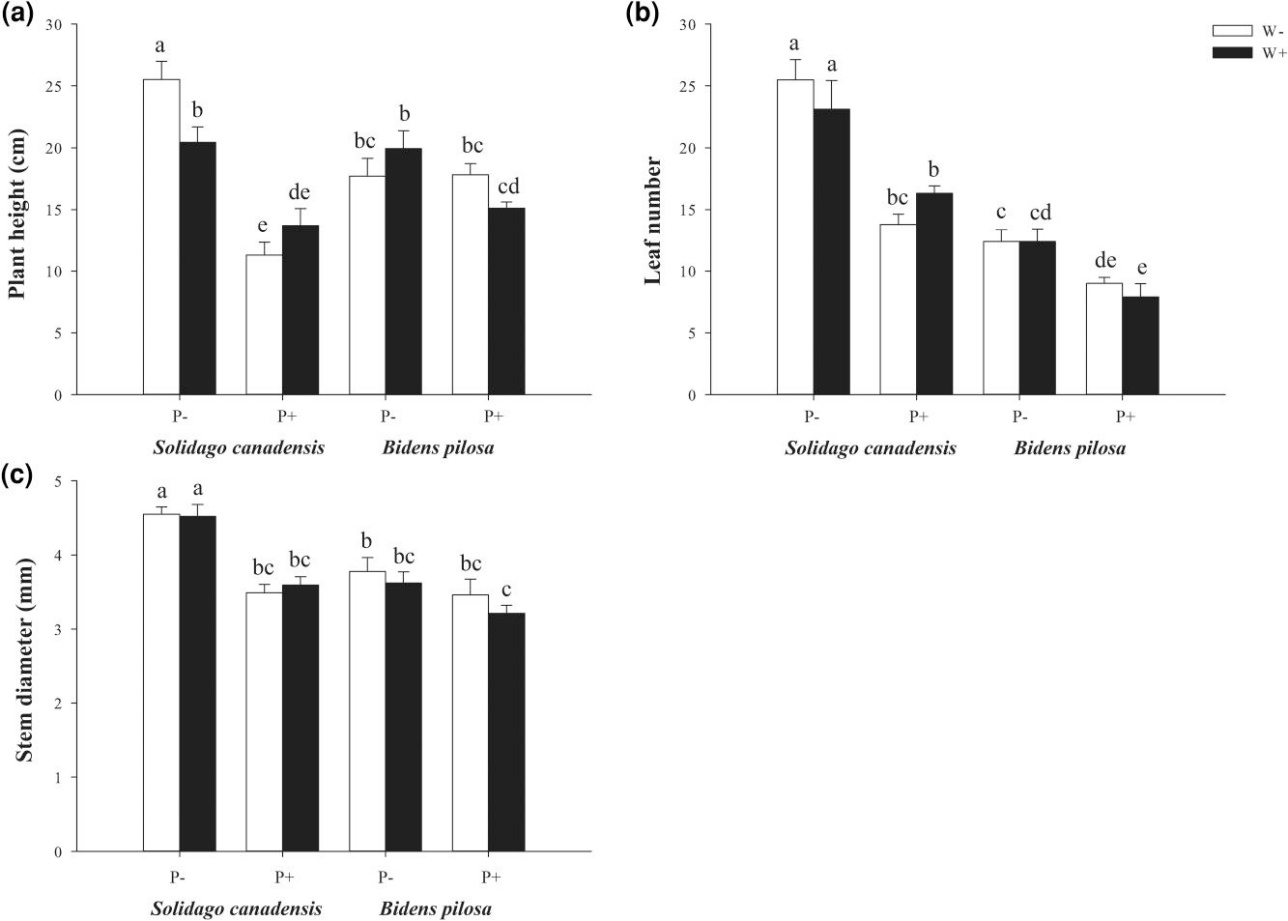
Effect of warming and *C. gronovii* parasitism on the (a) plant height, (b) leaf number, (c) stem diameter of the invasive plant species *Solidago canadensis* and *Bidens pilosa*. Values are means ± SE. Different letters indicate significant differences among treatments based on LSD *post hoc* tests (*P* < .05). W+: warming present, W−: warming absent, P+: parasitized by *C. gronovii*, P−: non-parasitized.

At ambient temperatures, *C. gronovii* parasitism significantly inhibited the growth of *S. canadensis* across all measured traits (Table 1; Figs 1 and 2). Total biomass decreased by 44%, with aboveground biomass decreasing by 37% and belowground biomass by 59%. Leaf number decreased by 46%, stem diameter by 23%, and plant height by 56%. However, elevated temperature altered these parasitism effects by modifying *S. canadensis*’s growth performance (Figs 1 and 2). Specifically, warming independently suppressed the growth of non-parasitized *S. canadensis* (total biomass decreased by 14%, and plant height by 20%). However, it mitigated the parasitic inhibition on most traits. Reductions in total biomass caused by parasitism were alleviated to 29% (15% alleviation), in belowground biomass by 36% (23% alleviation), in aboveground biomass by 26% (11% alleviation), in leaf number by 29% (17% alleviation), in stem diameter by 20% (3% alleviation), and in plant height by 33% (23% alleviation).

**Table 1:** Effect of warming (W), host species (H), *C. gronovii* parasitism (P), and their interactions on the growth of invasive plants.

Source of variation	d.f.	Total biomass		Aboveground biomass		Belowground biomass		Root:shoot ratio		Plant height		Leaf number		Stem diameter	
		*F*	*P*	*F*	*P*	*F*	*P*	*F*	*P*	*F*	*P*	*F*	*P*	*F*	*P*
P	1	149.678	**<**.**0001**	96.3658	**<**.**0001**	53.1919	**<**.**0001**	2.12788	.1499	54.3025	**<**.**0001**	66.7155	**<**.**0001**	40.498	**<**.**0001**
W	1	0.736	.4539	0.0221	.8912	0.9513	0.4013	0.11357	.7583	0.3022	.6208	0.0139	0.9136	0.2	.6848
H	1	100.758	**<**.**0001**	17.585	.**0001**	128.874	**<**.**0001**	61.2522	**<**.**0001**	0.0124	.9119	161.867	**<**.**0001**	23.309	**<**.**0001**
P × W	1	0.012	.9143	0.5967	.4429	0.8373	.3639	2.30303	.1345	0.2418	.6247	0.8887	.3497	0.001	.9787
P × H	1	0.265	.6087	1.4921	.2267	0.7947	.3763	11.19402	.**0014**	23.5892	**<**.**0001**	3.6352	.0614	8.379	.**0053**
W × H	1	1.049	.3098	1.3938	.2425	0.0075	.9314	0.43556	.5118	0.1556	.6946	1.5793	.2138	1.39	.2431
P × W × H	1	11.267	.**0014**	7.675	.**0075**	3.0049	.0882	0.02363	.8784	13.0872	.**0006**	4.6414	.**0353**	0.28	.5988

Bold *P* values indicate *P* < .05.

Unlike *S. canadensis*, *B. pilosa* exhibited distinct responses to parasitism and warming (Table 1; Figs 1 and 2). At ambient temperature, *C. gronovii* parasitism significantly reduced *B. pilosa*’s total biomass by 23%, its aboveground biomass by 25%, and its belowground biomass by 20%. Leaf number decreased by 27%. Parasitism had no significant effect on the stem diameter of *B. pilosa* at ambient temperatures but significantly decreased its plant height by 24% and increased the root-to-shoot ratio by 32% at elevated temperatures. Elevated temperature promoted the growth of non-parasitized *B. pilosa* (total biomass increased by 10% and aboveground biomass by 16%) but exacerbated parasitic inhibition of key traits (Figs 1 and 2). Parasitism reduced total biomass by 23% under ambient temperature and by 36% under warming temperature (an exacerbation of 13%) and reduced aboveground biomass by 25% under ambient temperature and by 41% under warming temperature (an exacerbation of 16%). Parasitism increased inhibition slightly for belowground biomass and leaf number (from 20% to 27% and from 27% to 36%, respectively), while the root-to-shoot ratio increased further due to the combined effects of parasitism and warming.

Host species identity significantly affected all growth traits (Table 1), including total biomass (*P* < .0001), aboveground biomass (*P* = .0001), belowground biomass (*P* < .0001), leaf number (*P* < .0001), stem diameter (*P* < .0001), and root-to-shoot ratio (*P* < .0001), indicating they differed substantially between *S. canadensis* and *B. pilosa*. Significant interactions were detected between parasitism and host species for stem diameter (*P* = .0053), plant height (*P* < .0001), and root-to-shoot ratio (*P* = .0014), confirming species-specific parasitic effects (Table 1; Figs 1 and 2). More importantly, the three-way interaction (parasitism × host species × warming) was significant for total biomass (*P* = .0014), aboveground biomass (*P* = .0075), leaf number (*P* = .0353), and plant height (*P* = .0006) (Table 1).

### Effects of elevated temperature and host species on the biomass of *C. gronovii* and its DEs on the host

**Figure 3:**
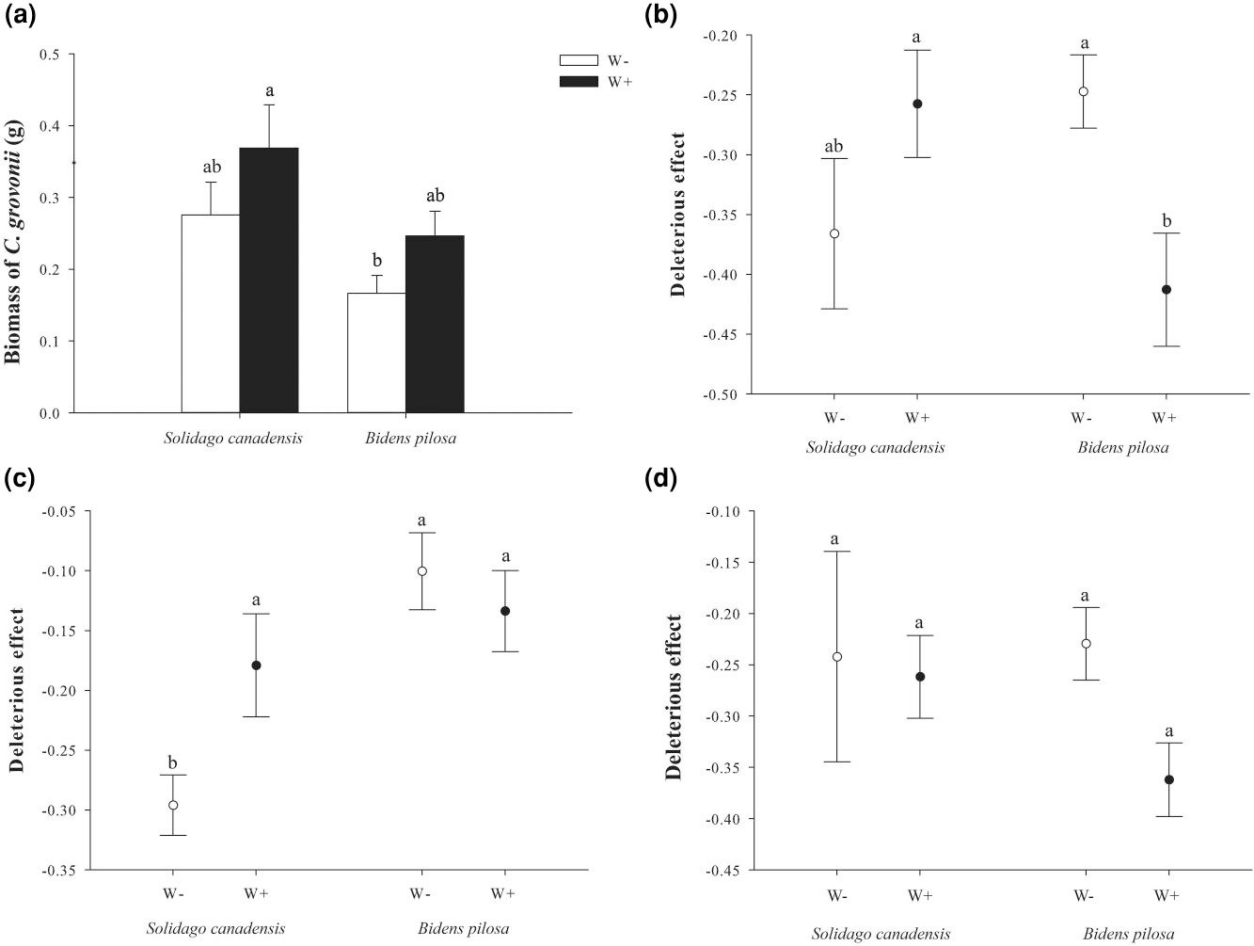
Detrimental effects of *C. gronovii* parasitism on the invasive plant species *Solidago canadensis* and *Bidens pilosa*. (a) The biomass of *Cuscuta gronovii*, (b–d) the deleterious effects of parasitism calculated from aboveground biomass, belowground biomass, and total biomass, respectively. Values are means ± SE. W+: warming present, W−: warming absent.

The elevated temperature did not significantly affect the biomass (*P* = .1537) of *C. gronovii* (Table 2; Fig. 3a). Host species significantly affected the biomass (*P* = .0096) of *C. gronovii*, but there was no significant interaction between elevated temperature and host species (*P* = .8038) (Table 2; Fig. 3a).

**Table 2:** Effect of warming (W), host species (H), and their interactions on the biomass of *C. gronovii* and its detrimental effects on the host.

Variables	Source of variation	d.f.	*F*	*P*
*C. gronovii* biomass	W	1	3.60863	.1537
	H	1	7.72097	**.0096**
	W × H	1	0.06288	.8038
Deleterious effects_T	W	1	1.82304	.2698
	H	1	0.77162	.3872
	W × H	1	1.01676	.3219
Deleterious effects_A	W	1	0.35979	.5909
	H	1	0.28684	.5965
	W × H	1	8.27379	**.0076**
Deleterious effects_B	W	1	1.17424	.3578
	H	1	21.71045	**.0001**
	W × H	1	8.38465	**.0073**
Deleterious effects_L	W	1	0.41308	.5662
	H	1	0.63346	.4328
	W × H	1	7.59669	**.0102**
Deleterious effects_H	W	1	20.549223	**.0201**
	H	1	0.542835	.4674
	W × H	1	0.485175	.4918
Deleterious effects_S	W	1	0.00017	.9905
	H	1	10.86096	**.0027**
	W × H	1	0.64678	.428

Bold *P* values indicate *P* < .05.

Deleterious effects_T: calculated based on total biomass; Deleterious effects_A: calculated based on aboveground biomass; Deleterious effects_B: calculated based on belowground biomass. Deleterious effects_L: calculated based on leaf number; Deleterious effects_H: calculated based on plant height; Deleterious effects_S: calculated based on stem diameter.

Elevated temperature did not significantly affect the DE of parasitism on *S. canadensis* based on aboveground biomass while significantly enhanced that on *B. pilosa* (Table 2; Fig. 3b). Conversely, elevated temperature significantly reduced the DEs of parasitism on *S. canadensis* based on belowground biomass, while no significant impact was observed for *B. pilosa* (Table 2; Fig. 3c). Furthermore, elevated temperature did not significantly affect the DE of parasitism on either *S. canadensis* or *B. pilosa* based on total biomass (Table 2; Fig. 3d). Host species significantly affected the DE of parasitism based on belowground biomass, and there was a significant interaction between elevated temperature and host species (Table 2; Fig. 3b and c). The DE of parasitism on leaf number was not affected independently by warming or host species, but their interaction was significant (Table 2). In contrast to other metrics, elevated temperature significantly enhanced the DE of parasitism on plant height (Table 2). Furthermore, host species significantly influenced the DE of parasitism on stem diameter. Specifically, it was observed that the negative effect of parasitism on stem diameter was significantly more pronounced in *S. canadensis* compared to *B. pilosa*, regardless of the temperature treatment administered (Table 2).

## Discussion

The interaction between climate warming and parasitic plants plays a crucial role in understanding the dynamics of invasive species and the efficacy of biological control measures. Our study reveals that a 2°C increase in ambient temperature significantly impacts the growth of invasive plants, with varying responses observed between *S. canadensis* and *B. pilosa*. This discrepancy highlights the complex nature of species-specific responses to environmental changes.

Warming can significantly affect the performance of invasive plants (Liu et al. 2017, Cao et al. 2018, Calvo et al. 2019, Bao et al. 2022, Ren et al. 2022, Sun et al. 2022, Yang et al. 2022), but the extent of its impact is contingent upon species and environmental contexts (Wang et al. 2019). In this study, we found that elevated temperature significantly inhibited the growth of *S. canadensis* but significantly enhanced the growth of *B. pilosa*. Similar phenomenon was reported by Yang (2023). These contrasting outcomes may be attributed to differences in life history traits. As a perennial plant, *S. canadensis* may allocate more resources to long-term survival mechanisms, making it more vulnerable to rapid environmental changes. As an annual plant, *B. pilosa* may exhibit strong plasticity in responding to rapidly changing environments, allowing it to rapidly undergo plastic adaptation (i.e. a phenotypic plastic response) to altered conditions, potentially via enhanced photosynthetic efficiency (Yamori et al. 2014). Further studies should evaluate the general principle regarding the effect of elevated temperatures by examining a wider range of species with diverse life histories.

Parasitic plants absorb water and nutrients from host plants, significantly affecting host plant vigour and growth (Pennings and Callaway 2002, Press and Phoenix 2005). Our study demonstrates that parasitism by *C. gronovii* negatively affects the growth parameters in both *S. canadensis* and *B. pilosa*. These findings are consistent with earlier research that documented comparable negative effects instigated by other parasitic species, such as *C. japonica* (Jiang *et al.* 2008), *C. australis* (Wang 2010, Li et al. 2012, 2015, Yang et al. 2015, Gao et al. 2021, Yuan et al. 2023), and *C. chinensis* (Wang 2010). While previous studies have focused on the impact of parasitism on host plants, our research emphasizes the pivotal role of climate warming in shaping these interactive dynamics. This investigation is vital for understanding how environmental changes may alter the effectiveness of parasitic plants in controlling invasive species under projected climatic conditions.

When analyzing the effects of elevated temperatures and parasitism on the two invasive species separately, a significant interaction between parasitism and elevated temperature was observed (Supplementary Tables S1 and S2). Specifically, elevated temperature significantly decreased *S. canadensis* total biomass, belowground biomass, and plant height under parasitism-free conditions. In contrast, elevated temperature increased these growth metrics under parasitism, although this increase was not statistically significant. Regarding *B. pilosa*, elevated temperature significantly increased the aboveground biomass under parasitism-free conditions but decreased plant height under parasitism. The observed differences in the responses of *S. canadensis* and *B. pilosa* to parasitism at elevated temperatures may be attributed to differences in heat tolerance. *S. canadensis* has a lower heat adaptation threshold, and warming intensifies the resource limitation (e.g. water and nutrients) imposed by parasitism (Xu et al. 2020). In contrast, *B. pilosa* exhibits stronger heat plasticity, enabling it to partially offset the effects of parasitism by adjusting its photosynthetic efficiency (Yamori et al. 2014, Chauhan et al. 2019). Consequently, in the combined analysis of these two species, no significant interactions were observed regarding the effects of elevated temperatures and parasitism. The overall interaction between elevated temperature and parasitism varied significantly among the different species examined, emphasizing the importance of conducting species-specific analyses to better understand the impacts of climate change and biotic interactions on invasive plant communities. This divergence in growth strategies suggests that the effectiveness of *C. gronovii* as a biological control agent may depend on the host species and environmental conditions.

Notably, elevated temperatures did not mitigate parasitism’s DE on invasive plants. This observation may be attributed to the positive effect of elevated temperatures on the compensatory growth mechanisms of host plants under parasitism. Specifically, our findings on *S. canadensis* revealed a significant occurrence of compensatory growth in response to parasitism, whereas such a phenomenon was absent in *B. pilosa*. This discrepancy is consistent with the significant interactions among elevated temperatures, parasitism, and host species, suggesting that the ability of host plants to compensate for parasitism-induced stress under elevated temperatures varies significantly depending on the species involved. In natural environments, plants encounter inherent resource limitations that constrain their various physiological processes, making it often impossible for plants to simultaneously meet all their requirements, leading to resource-based trade-offs among various functions (Herms and Mattson 1992, Züst and Agrawal 2017). This also occurs when plants encounter parasitic plants. For example, Zhang et al. (2012) identified a compensatory growth mechanism of the host *B. pilosa* under high parasitism pressure from *C. australi*s. Previous research has shown that elevated temperatures can enhance the host’s root:shoot ratio during hemiparasitic interactions (Rafferty et al. 2019). Consistent with these findings, the significant increase in the root:shoot ratio of *B. pilosa* under parasitism and warming conditions indicates a potential adaptive strategy to mitigate the DEs of parasitism. Enhanced root growth can improve *B. pilosa*’s ability to acquire water and nutrients from the soil, countering the pressure of parasitism. So, elevated temperature significantly enhanced the DE of parasitism on *B. pilosa* based on aboveground biomass, while no significant impact was observed based on belowground biomass. In contrast, elevated temperature did not affect *S. canadensis* root:shoot ratio irrespective of *C. gronovii* parasitism, suggesting that the resource acquisition ability of *S. canadensis* remained unaffected by the interaction between parasitism and elevated temperatures. Thus, elevated temperature had no significant effect on the DE of *C. gronovii* parasitism on *S. canadensis* when assessed by aboveground biomass. However, it significantly alleviated this DE when evaluated by belowground biomass. Future research should focus on the mechanisms underlying the species-specific responses to parasitism and climate warming. This should include an examination of physiological traits such as photosynthetic efficiency, resource allocation patterns, and adaptive growth strategies.

To balance the biocontrol effect on invasive plants with the protection of native plants, we recommend that future biocontrol practices adopt habitat-specific release strategies. These strategies should prioritize the release of *C. gronovii* in areas with a high density of invasive plants (e.g. *S. canadensis* and *B. pilosa*), in order to minimize contact with native plants. This study notably provides a foundation for understanding *C. gronovii*’s interactions with specific invasive plant species, but further research is needed to quantify its non-target effects on diverse native plant communities in different ecosystems. Long-term field experiments tracking the dynamics of *C. gronovii*, invasive plants, and native plants are critical to the refinement of safe and effective biocontrol strategies in the context of climate change. Additionally, long-term field monitoring of *C. gronovii* population dynamics and its host range is necessary to assess ecological risks and adjust biocontrol strategies in a timely manner. Further studies could quantify the host preference of *C. gronovii* for invasive versus native plants, providing a scientific basis for ecological safety in biocontrol. Experimental studies across a broader range of invasive species and parasitic plants are essential to determine the generalizability of these findings. Such research is crucial for developing robust biological control strategies that can mitigate the impact of invasive species in the context of projected climate change.

## Conclusions

This study sheds new light on how elevated ambient temperatures influence the response of invasive plants to biological control agents, namely holoparasitic plants. Our key findings are as follows: (i) Elevated temperature (2°C) does not exert a consistent inhibitory effect on *S. canadensis* or promotional effect on *B. pilosa* across both parasitized and non-parasitized conditions. (ii) The effect of warming on *C. gronovii’s* parasitic harm is species-specific: it alleviates the belowground biomass loss in *S. canadensis* and exacerbates the aboveground biomass loss in *B. pilosa*. These species-specific responses may be due to the hosts’ life history traits (perennial versus annual) and their resource allocation strategies. Further research will be conducted to test the effect of life history traits on the warming responses using multiple species experiments. These findings emphasis the intricate relationship between parasitism, host species, and environmental factors, all of which are essential for a comprehensive understanding of the dynamics of invasive species dynamics and their ecological implications. The species-specific nature of the host plants’ response to holoparasitic plants emphasizes the need for future research to consider a wider range of invasive plant species. This will improve our understanding of these interactions and provide valuable information on the potential effectiveness of parasitic plants as biocontrol agents in the context of climate change.

## Supplementary Material

plag015_Supplementary_Data

## Data Availability

Raw data and R code are available online at https://zenodo.org/records/18645262.
