# Supplementary material for: Species-specific responses of invasive plants to parasitism are modified by climate warming
Source: AoB Plants. 2026 Feb 27;18(2):plag015. doi: 10.1093/aobpla/plag015 (PMC12982917; doi:10.1093/aobpla/plag015)
Supplement: plag015_Supplementary_Data [file plag015_supplementary_data.pdf]

Species-specific responses of invasive plants to parasitism are modified by climate warming

This file includes:

Tables

Table S1. Effect of warming (W), and *C. gronovii* parasitism (P) on the growth of *Solidago canadensis*. Bold P values indicate P < 0.05.

Table S2. Effect of warming (W), and *C. gronovii* parasitism (P) on the growth of *Bidens pilosa*. Bold P values indicate P < 0.05.

Tables

Table S1. Effect of warming (W), and *C. gronovii* parasitism (P) on the growth of *Solidago canadensis*. Bold *P* values indicate $P < 0.05$.

Variables	Source of variation	d.f.	*F*	*P*
Total biomass	P	1	74.491	**<.0001**
	W	1	1.0473	0.3815
	P × W	1	5.3685	**0.0286**
Aboveground biomass	P	1	44.8953	**<.0001**
	W	1	0.6209	0.4882
	P × W	1	1.7479	0.1977
Belowground biomass	P	1	49.4574	**<.0001**
	W	1	0.0192	0.8986
	P × W	1	4.5902	**0.0417**
Root: shoot ratio	P	1	9.7435	**0.0044**
	W	1	0.0022	0.9658
	P × W	1	1.6067	0.2162
Plant height	P	1	79.2560	**<.0001**
	W	1	0.7626	0.4468
	P × W	1	9.18885	**0.0055**
Leaf number	P	1	5.38828	**0.0284**
	W	1	0.79063	0.4394
	P × W	1	0.03009	0.8636
Stem diameter	P	1	62.243	**<.0001**
	W	1	0.127	0.7455
	P × W	1	0.278	0.6024

Table S2. Effect of warming (W), and *C. gronovii* parasitism (P) on the growth of *Bidens pilosa*. Bold *P* values indicate *P* < 0.05.

Variables	Source of variation	d.f.	*F*	*P*
Total biomass	P	1	78.5327	**<.0001**
	W	1	0.1701	0.7077
	P × W	1	5.8085	**0.023**
Aboveground biomass	P	1	56.6503	**<.0001**
	W	1	0.8241	0.4309
	P × W	1	5.9579	**0.0215**
Belowground biomass	P	1	13.3084	**0.0011**
	W	1	0.1844	0.6966
	P × W	1	0.2783	0.6021
Root: shoot ratio	P	1	2.08484	0.1603
	W	1	0.32312	0.6095
	P × W	1	1.12348	0.2986
Plant height	P	1	4.28	**0.0483**
	W	1	0.13	0.7427
	P × W	1	6.153	**0.0196**
Leaf number	P	1	18.2474	**0.0002**
	W	1	0.3868	0.5781
	P × W	1	0.3323	0.5691
Stem diameter	P	1	5.8192	**0.0229**
	W	1	1.5211	0.3053
	P × W	1	0.0688	0.795
